# Emerging applications of cancer bacteriotherapy towards treatment of pancreatic cancer

**DOI:** 10.3389/fonc.2023.1217095

**Published:** 2023-07-19

**Authors:** Emily A. Henderson, Slawomir Lukomski, Brian A. Boone

**Affiliations:** ^1^ Department of Microbiology, Immunology and Cell Biology, West Virginia University, Morgantown, WV, United States; ^2^ West Virginia Cancer Institute, West Virginia University, Morgantown, WV, United States; ^3^ Department of Surgery, West Virginia University, Morgantown, WV, United States

**Keywords:** bacterial therapy, pancreatic ductal adenocarcinoma, immunotherapy, anti-tumor immunity, bioengineering

## Abstract

Pancreatic cancer is a highly aggressive form of cancer with a five-year survival rate of only ten percent. Pancreatic ductal adenocarcinoma (PDAC) accounts for ninety percent of those cases. PDAC is associated with a dense stroma that confers resistance to current treatment modalities. Increasing resistance to cancer treatments poses a challenge and a need for alternative therapies. Bacterial mediated cancer therapies were proposed in the late 1800s by Dr. William Coley when he injected osteosarcoma patients with live streptococci or a fabrication of heat-killed *Streptococcus pyogenes* and *Serratia marcescens* known as Coley’s toxin. Since then, several bacteria have gained recognition for possible roles in potentiating treatment response, enhancing anti-tumor immunity, and alleviating adverse effects to standard treatment options. This review highlights key bacterial mechanisms and structures that promote anti-tumor immunity, challenges and risks associated with bacterial mediated cancer therapies, and applications and opportunities for use in PDAC management.

## 1 Introduction

Pancreatic cancer is one of the most lethal cancers with an average five-year survival rate of only ten percent (1, 2). Pancreatic ductal adenocarcinoma (PDAC) accounts for over ninety percent of pancreatic cancer cases and is the fourth leading cause of cancer death in the United States (2, 3). Current treatment plans of PDAC consist of surgical resection, when possible, and combination chemotherapy of gemcitabine and nab-paclitaxel or FOLFIRINOX. However, a majority of patients are not eligible for surgery at presentation due to metastases or local invasion of mesenteric vessels. Furthermore, most post-surgical patients develop local or metastatic recurrence despite margin negative resection. Despite recent advances in chemotherapy, these regimens still offer poor survival benefit, improving survival only 3-4 months in the setting of metastatic disease (4).

Several factors influence the poor prognosis seen in PDAC. Pancreatic cancer is typically diagnosed at later stages due to propensity for early metastatic spread and late symptom onset (2). Moreover, PDAC is associated with a dense stroma of desmoplasia comprised of fibroblasts and inflammatory cells. Pancreatic stellate cells exhibit fibroblast characteristics and contribute to the desmoplastic response that promotes the aggressive progression of PDAC. Pancreatic stellate cells are the predominant source for the extracellular matrix (ECM) in this unique tumor microenvironment (TME), which makes targeted therapies challenging by reducing the effectiveness of drug delivery and infiltration of anti-tumor immune cells (3, 5). These factors pose challenges for treatment and improving patient outcomes.

While immunotherapy and particularly checkpoint therapy has revolutionized the management of many cancers, treatment responses in PDAC have been minimal. A phase II clinical trial (AstraZeneca, NCT02583477), for example, investigated the benefit of Durvalumab, a monoclonal antibody that blocks programmed cell death ligand-1 (PDL-1), combined with additional cancer drugs for metastatic PDAC. There was no change in tumor shrinkage and a significant percentage of patients exhibited adverse events (6). Another trial examined the efficacy of the combination therapy of Paclitaxel and Gemcitabine with Demcizumab (OncoMed Pharmaceuticals, Inc., NCT02289898) in metastatic PDAC. While overall survival of participants increased, there is still little evidence to survival differences over FOLFIRINOX therapy alone (7, 8). This lack of response to immunotherapy is due to several factors attributed to the unique PDAC TME. The dense, fibrotic stroma prevents infiltration of T cells into the tumor. Activated cancer associated fibroblasts promote an immunosuppressive milieu that diminishes immune cell anti-tumor response and increase myeloid derived suppressor cells and T regulatory cells. Furthermore, PDAC is characterized by vast genetic heterogeneity. KRAS mutations account for about 90% of invasive cancers. CDKN2A, TP53, and SMAD4 mutations contribute to the inactivation of tumor suppressors (1, 3, 9–12). However, despite these genetic drivers, there are a paucity of neoantigens which reduces immune activation in response to tumor. As a result, research and development of novel therapeutics that target the PDAC TME and activate the response to immunotherapy is critical to improving outcomes for this devastating disease.

One therapeutic approach that has been resurrected from its historical use is utilizing bacteria or bacterial components – such as “Coley’s toxin” - as a cancer immunomodulatory agent (13). Although potentially harmful in the setting of infection, many bacteria can be engineered for therapeutic value in cancer, offering a range of targetable components varying from toxins to mechanical structures such as flagella. This review focuses on the history of bacteria-derived therapeutics, the molecular mechanisms of bacteria or bacterial products as potential therapies, and the outlook for bacteria in cancer research and immunotherapy development in PDAC.

## 2 Historical perspective on cancer bacteriotherapy

**Figure 1:**
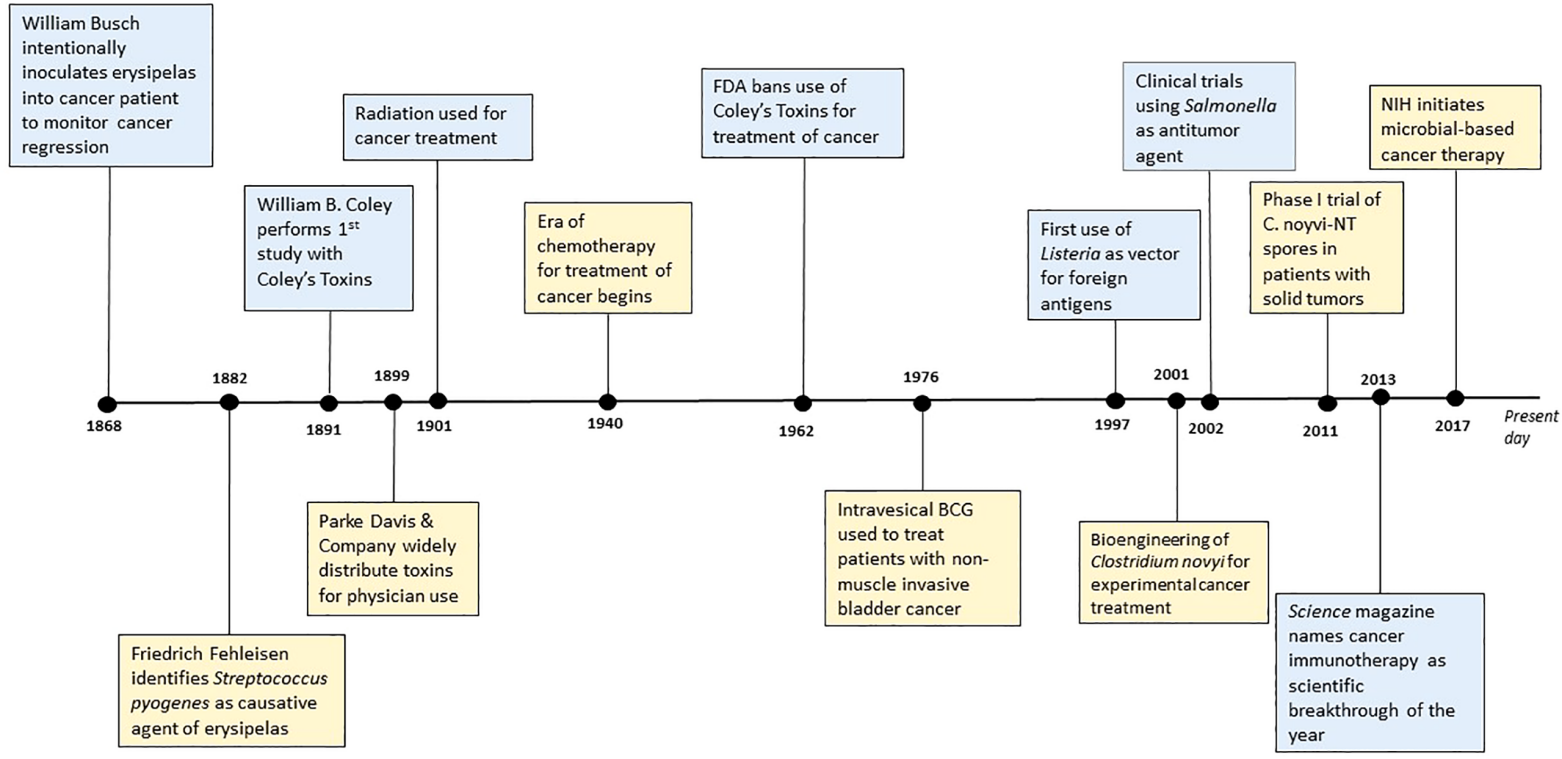
Historical timeline of significant contributions to bacterial-mediated cancer therapy.

The effect of bacteria on cancer progression has been documented for decades; **Figure 1** depicts a general timeline of important therapeutic contributions. In the late 1800s, German physicians Wilhelm Busch and Friedrich Fehleisen noted cancer regression in patients following erysipelas infections, with the latter identifying *Streptococcus pyogenes* as the causative agent of erysipelas (13, 14). Their work motivated the systematic cancer treatments performed by American surgeon William B. Coley. In 1891, Coley performed his first study of cancer bacteriotherapy by injecting live *S. pyogenes* into three patients and causing deadly infection in two of the patients. To mitigate the harmful effects of infection, Coley used a mix of heat-killed *S. pyogenes* and *Serratia marcescens –* a combination known as Coley’s Toxin (14, 15). Coley administered this treatment to hundreds of patients with inoperable bone and soft-tissue sarcomas. By 1899, the pharmaceutical company Parke Davis & Company began to manufacture and distribute Coley’s toxins for physician use where they were widely used for the next thirty years. However, Coley was criticized for his work. There was variability in his methods of administration and differences between toxins’ preparations, which was compounded by a long unsafe product transport overseas, all resulting in contrasting results when utilized by other doctors. The use of radiation therapy and chemotherapy gained more favor and gradually Coley’s toxins disappeared from use in 1952. In 1962, the Food and Drug Administration (FDA) banned the use of Coley’s toxins for the treatment of cancer altogether. Still, Coley’s pioneering contributions paved the way for bacterial immunotherapy (14, 16, 17).

Since Coley, scientists have explored a variety of bacterial-mediated therapies for the treatment of cancer. One of the most noteworthy still used today is the installation of bacille Calmette-Guerin (BCG) bacilli to treat superficial bladder cancer (14, 16, 18, 19). In 1921, the BCG vaccine developed by Calmette and Guerin began its clinical use against tuberculosis infection. However, in 1929, it was noted that patients with tuberculosis had lower rates of cancer. This led to a study published in 1976 by Morales et al. reporting intravesical inoculation of BCG to be an effective therapy for non-muscle-invasive bladder cancer (19–23). This is still considered to be one of the most successful treatments for superficial bladder cancer (22, 24) and perhaps one of the most significant bacterial-mediated cancer therapies. Throughout the rest of the 20^th^ century up to present day, a variety of bacteria have been distinguished as potential therapeutic agents. *S. pyogenes*, *Bifidobacterium* spp.*, Clostridium* spp.*, Listeria monocytogenes*, and *Salmonella typhimurium* are just a few. Both *Clostridium* spp. and *S. typhimurium* in combination with chemotherapeutic agents or radiation show promising results (17, 25). The *Salmonella* strains VNP20009 and AR-1 combined with hydroxychloroquine or gemcitabine, respectively, show promising results in breast and pancreatic cancer. Moreover, both *Salmonella* VNP20009 and *Clostridium novyi* – nontoxic - are being evaluated for phase I clinical trials in humans (17, 25). The rapid development and advancement in molecular technology gives us the ability to exploit and modify various molecular mechanisms governed by microbes to our advantage. These technologic advances have unveiled several potential non-infectious strategies that could make bacterial immunotherapy more appealing to patients and clinical providers. These anti-tumor mechanisms will be discussed in later sections.

## 3 Bacterial induction of immune response and antitumor effects

**Figure 2:**
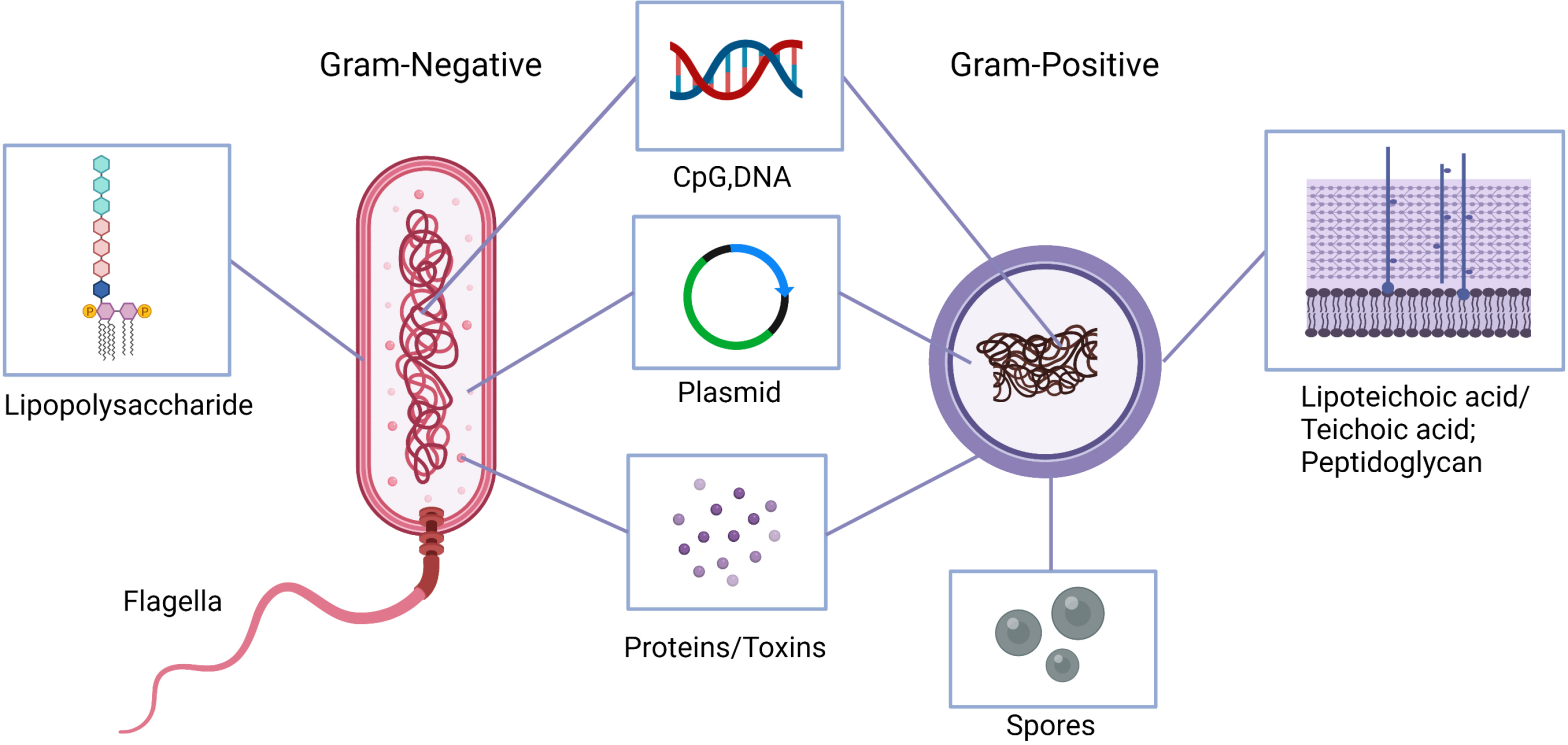
Natural and engineered bacterial components with anti-tumor activities. Lipopolysaccharide or lipoteichoic acid and peptidoglycan, flagella, bacterial DNA, as well as synthetic CpG oligodeoxynucleotides stimulate the immune system to produce cytokines to activate antigen presenting cells and induce T-helper 1 responses. Bacterial proteins can induce anti-tumor immune responses as well as inhibit inflammatory and pro-tumor responses. Engineered anaerobic bacteria, e.g., clostridia, outgrow from the injected spores within hypoxic tumor environment and produce local cytotoxicity. Similarly, bacterial plasmids are engineered to express tumor-associated antigens and enhance drug delivery.

**Figure 3:**
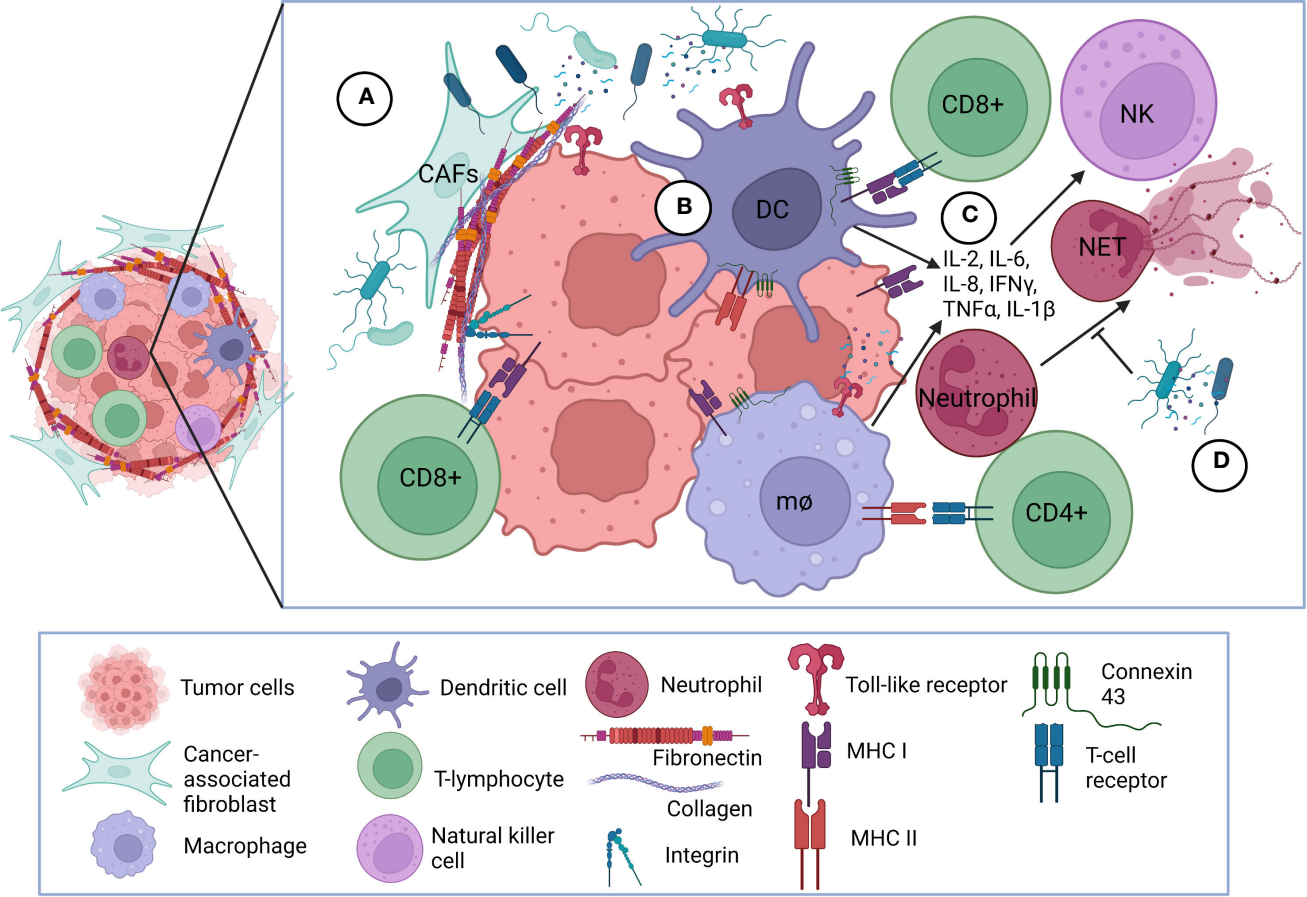
Bacterial interaction with tumor microenvironment. **(A)** Bacterial interactions with extracellular matrix and cellular receptors. Bacteria adhere to fibronectin and collagen deposited mostly by cancer-associated fibroblasts and forming the fibrotic extracellular matrix surrounding the tumor. Bacteria can also bind directly or via bridging mechanism mediated by fibronectin and collagen to selected integrins overexpressed on cancer cells facilitating internalization into the cancer cells. Bacterial components and peptides also bind to TLRs on the surface of cancer cells and antigen-presenting cells. **(B)** Bacteria initiate immune responses within tumor microenvironment. Bacteria and/or bacterial components become processed into peptides which are presented onto MHC class I or class II molecules on the surface of antigen presenting cells and cancer cells. CD8+ and CD4+ T-lymphocytes interact with MHC class I and II complexes, respectively, via TCRs. Upregulation of connexin-43 can also facilitate greater gap junction communication among immune cells to present tumor peptides to CD8+ T-lymphocytes. **(C)** Bacteria-initiated immune responses elicit inflammatory mediators. Following antigen-presentation, pro-inflammatory cytokines are released to further activate and attract CD8+ and CD4+ T-lymphocytes to the tumor and stimulate NK cell proliferation. **(D)** Bacteria modulate cancer-associated NETosis. Bacteria and bacterial products can inhibit NET formation and release; CAF, cancer-associated fibroblast; TLR, Toll-like receptor; MHC, Major Histocompatibility Complex; TCR, T-cell receptor; NK, natural killer; mθ, macrophage; NET, neutrophil extracellular trap; DC, dendritic cell.

Microorganism invasion and multiplication in host leads to a cascade of both innate and adaptive immunologic responses (26). This response is no exception in cancer. Bacteria attach to tumor cells and deploy mechanisms that elicit host immunity (19). While most of these mechanisms are still unclear, many studies demonstrate the importance of bacterial attachment, internalization, and immune system induction in effective cancer therapy. **Figures 2**, **3** highlight some of these significant bacterial features and mechanisms discussed in the following sections (27).

These functions can further be exploited to enhance drug efficacy and improve clinical outcomes. The overall mechanisms of bacterial mediated cancer therapy can be broken down into two major phases – the induction of host immunity and direct antitumor effects. Numerous bacteria have been evaluated as anti-cancer agents and hold therapeutic premise for pancreatic cancer. An overview of each bacterium is provided below discussing relevant data and anti-cancer mechanisms. **Table 1** summarizes the bacteria that have been specifically evaluated for bacterial mediated cancer therapies in pancreatic cancer. Although not all bacterial species have been specifically studied in PDAC treatment, the research findings gained from different cancer bacteriotherapies remain relevant and suggest targets for further exploration.

**Table 1:** Relevant clinical trials exploring bacterial mediated cancer therapy in PDAC.

Bacteria species	Innate Immune Responses	Anti-tumor Effects	Relevant Clinical Trials
*Salmonella typhimurium*	LPS-TLR4 Flagellin-TLR5 Enhance chemosensitivity of APCs via connexin 43 Inflammasome formation	Regulate apoptosis and autophagy Enhanced processing of tumor associated antigens (TAAs) Activation of cytotoxic CD8+ T cells	NCT04589234 (28)
*Listeria monocytogenes*	Listeriolysin O (LLO) Activation of STING pathway	Priming of CD8+ T cells against TAAs Induction of apoptosis in tumor cells Production of reactive oxygen species (ROS)	NCT03190265 (29) NCT05014776 (30) NCT01417000 (31) NCT02004262 (32)
*Lactobacillus* spp.	Flagellin-TLR5 Lipoteichoic Acids Modulate cytokine milieu	Enhanced NK cell activity T-cell polarization against tumor cells	No current clinical trials but shows promise in PDAC (33)

### 3.1 Bacillus Calmette-Guerin (*Mycobacterium bovis*)

As mentioned in the previous section, BCG has been used for decades in the treatment of superficial bladder cancer. Instillation of BCG into the bladder allows the bacteria to contact the urothelium and bladder cancer cells themselves on the luminal surface. The mycobacterial fibronectin attachment protein interacts with host fibronectin through integrin α5β1mediating attachment (19, 20, 34, 35). Fibronectin is a part of the extracellular matrix (ECM) distributed on normal and malignant urothelium. The fibronectin attachment protein of BCG has a high affinity receptor for the collagen domain of fibronectin stimulating the internalization of BCG into tumor cells. Fibronectin serves as a bridging molecule to both urothelial cells and BCG working with other molecules like heparin sulphate containing proteoglycans that interact with the mycobacterial heparin-binding hemagglutinin adhesin (20, 34). In other words, fibronectin is not mandatory but rather enhances adherence of BCG to the bladder wall. Data suggests that the addition of anti-fibronectin antibodies can impair BCG binding to the murine bladder. In addition, the clinical effects of BCG therapy can be related to the degree of fibronectin expression (34).

Following internalization, BCG is broken down into various peptides and proteins causing phenotypic alterations within the cancer and bladder cells that trigger the host immune system. These peptides are processed and associate with major histocompatibility complex (MHC) class II molecules that get expressed on the surface of professional antigen presenting cells (APCs) or MHC class I molecules on the surface of urothelial tumor cells (18, 20, 34, 35). The release of pro-inflammatory cytokines such as interleukin (IL)-1, IL-2, IL-6, IL-8, IL-12, tumor necrosis factor (TNF)-α, and interferon (IFN)-γ also occurs following antigen presentation. Cytokines cause further recruitment of immune cells such as CD8+ and CD4+ T lymphocytes, macrophages, and neutrophils to induce a Th1 cytotoxic response and enhance cancer cell recognition (20, 22, 34).

IL-2, IL-6, IL-8 and IFN-γ are particularly important because they can “fine tune” the generation of APCs and activation of T-cells which contribute to antitumor effects. IL-6 stimulates T cell proliferation, macrophage maturation, cytotoxic T-cell differentiation, and activation of resting T cells via interactions with IL-2 and IL-2 receptor expression. In cancer, IL-6 induces natural killer (NK) cell proliferation which contributes to the MHC-nonrestricted cytotoxic activity of those cells. Dendritic cells (DCs) and macrophages produce IL-8 which has strong chemotactic properties that attract T lymphocytes, neutrophils, and other innate effector cells to induce antitumor effects (24, 34).

More recently, scientists recognize the importance of IL-17. IL-17 induces neutrophil infiltration in the bladder producing chemokines such as MIP-1α and GRO-α for CD4+ T-cell migration and generates important Th1-cell responses (23, 34, 35). Luo et al. show that this Th1-cell response is vital for BCG treatment particularly to produce IFN-γ. While IFN-γ appears to be a late responder in the cytokine milieu, it has a role in driving the activation of CD8+ cytotoxic T lymphocytes (20, 36). CD4+ T-cells and cytotoxic CD8+ T cells mediate the Th1 cell response by IL-2, TNF, IL-12, and IFN-γ secretion. NK cells drive anti-tumor immunity through Th2 responses via IL-4, IL-5, IL-6, and IL-10. This immune cascade from these cytokines causes direct anti-tumor activity (20). The cytokine milieu during BCG therapy is of recent interest. There is evidence suggesting that urinary IL-2 levels as well as IL-6, IL-8, IL-12, TNF-α, and IFN-γ are promising markers in predicting BCG clinical responses (19, 22) suggesting cytokines as a potential treatment for cancer. High-dose IL-2 for example is approved for the treatment of metastatic renal cell cancer and melanoma with many others like IL-12 and IFN-γ under investigation (37). Since relapse typically occurs within the first five years of treatment with BCG (34), combination treatments with cytokines could enhance efficacy and side effect reduction.

### 3.2 *Streptococcus pyogenes* (GAS)

As discussed in the previous section, Coley and physicians over the years explored the use of *S. pyogenes* in the treatment of cancer. While Coley used heat-inactivated bacteria for his studies, live *S. pyogenes* has several adaptations that provide the possibility for future cancer treatments such as the selective recognition and attachment to oncofetal fibronectin via streptococcal collagen like protein 1 (Scl1) (38–40), as well as a variety of immunomodulatory proteins and enzymes.

The Scl1 protein is anchored to the cell wall of *S. pyogenes* in homotrimeric organization (40). It consists of a central collagen-like (CL) region comprised of Gly-Xaa-Yaa repeats, a non-collagenous N-terminal V-domain, and a C-terminal cell wall-associated region (38, 39). The CL region and V-domain have significant implications in cancer. The central CL domain allows for a triple helical formation that mimics that of mammalian collagen. This similarity supports direct interactions with collagen binding integrins such as α_2_β_1_ and α_11_β_1_ facilitating internalization by human cells (38, 40–42). The V-domain is important in the stabilization of a triple helical conformation of the CL domain (43, 44). It directs Scl1 recognition and binding to the cellular fibronectin type III repeats, the extracellular domains A (EDA) and B (EDB) – also known as oncofetal fibronectin – deposited by cancer associated fibroblasts (CAFs) (39, 40, 45, 46). Solid tumors have extensive ECM deposition due to stromal CAFs (47–49). These CAFs deposit EDA/EDB-containing fibronectins in the tumor microenvironment (TME) where Scl1 mediates adhesion by GAS (39).

There is some indication that Scl1 may modulate other aspects of host immunity such as neutrophil infiltration and neutrophil extracellular trap formation. Neutrophil extracellular traps (NETs) have gained recognition for their role in inflammatory disease and cancer. NETs occur when activated neutrophils release their intracellular contents including DNA, histones and granules into the tumor microenvironment or circulation. NETs can promote the migration and invasion of cancer cells, mediate the epithelial-mesenchymal transition, awaken dormant cancer cells, and facilitate metastasis (50–52). While NETs are detrimental in cancer progression, they provide necessary protection against bacterial infection (53). However, bacteria deploy various virulence factors to evade NETs. *S. pyogenes* has several mechanisms for this (54–56). While limited research shows the specific mechanistic role of Scl1 in NET inhibition, Dohrmann et al. show that *scl1* mutant strain of GAS is more susceptible to phagocytic killing by neutrophils and induce more NETs in comparison to wild-type or complemented strains. Evidence from this study suggests that surface Scl1 can hinder the release of myeloperoxidase (MPO) from NETs and directly protect bacteria from cathelicidin LL-37, a molecule that induces pore formation in bacterial membranes (54).

The M1 protein of GAS can elude NET killing by promoting proinflammatory conditions and evading phagocytosis. M proteins facilitate GAS adherence to fibronectin which can cause an immune cascade of neutrophil infiltration leading to NET release. However, the M1 protein confers resistance to NET killing by cathelicidin LL-37 release following phagocytic degranulation (57). GAS strains also secrete streptolysin O (SLO), a toxin belonging to the cholesterol-dependent cytolysins family that promotes resistance to neutrophil clearance (56). SLO binds to the cholesterol lipid rafts in the eukaryotic cell membrane and forms large pores. Typically, this is not cytocidal unless in large quantities (58). Rather, SLO blocks neutrophil oxidative burst and IL-8 responsiveness leading to resistance of neutrophil killing (56). Although the benefits of SLO in cancer responsiveness are limited, Hall et al. report that SLO can reduce breast cancer invasion and cell growth by activating the ErbB1 kinase, a protein encoded by the epidermal growth factor receptor gene involved in cell signaling and proliferation (59). Therefore, future cancer studies involving SLO and molecular signaling will be beneficial.

DNase expression is another way that bacteria, including GAS, can escape neutrophil killing. DNase Sda1 is a secreted endonuclease that provides protection against the host innate immune response. Specifically, Sda1 can degrade the DNA component of NETs as well as that seen in the extracellular traps of macrophages. Evidence suggests that Sda1 may suppress the TLR9-mediated immune response by degradation of CpG-rich DNA (55, 60). Therefore, targeting NETs via bacterial mechanisms such as a DNase-based therapy may be a useful approach in inhibiting NET formation and release during cancer.

### 3.3 Salmonella typhimurium

*S. typhimurium* is extensively studied for its potential application in cancer treatment. For one, *S. typhimurium* is a facultative anaerobe, allowing it to adapt to both the hypoxic and nonhypoxic zones within a tumor. Second, proper flagella constructs, active motor function, or signal transduction proteins are necessary for tropism to the tumor region where the bacterium can encounter an aspartate receptor, serine receptor, or a galactose or ribose receptor (25). From here, *S. typhimurium* uses a type-3 secretion system to infect the cancerous cells, colonize the tumor, and activate antitumor immunity (25, 61). However, the tumor cells are not directly destroyed by the bacteria in this manner. Rather, antigen presenting cells (APCs) present bacterial antigens targeted by anti-salmonella T cells (61).

APCs can activate the adaptive immune response against *S. typhimurium* in different ways. Salmonella bacteria are gram negative with a lipopolysaccharide (LPS) on the outer membrane. LPS is a strong microbial associated molecular pattern (MAMP) molecule that can activate toll-like receptor 4 (TLR4), stimulating the release of different proinflammatory cytokines like IL-6, IL-8, TNF-α, and matrix metallopeptidase (MMP)-9 (27, 62). This overexpression of IL-6 plays a major role in inflammatory diseases and cancer (63). The bacterial flagellum can interact with TLR5 and mediate innate immunity in a similar way, causing elevated cytokine expression and subsequent activation of nuclear factor kappa B (NF-κB) and the Janus Kinase/STAT3 pathway, promoting the maturation of dendritic cells and enhancement of anti-tumor immunity (27, 62). The upregulation of pro-inflammatory cytokines can also lead to the production of IFNγ which can induce chemokines CXCL9 and CXCL10 to recruit NK cells, neutrophils, macrophages, and T cells to the tumor and stimulate acquired immunity for a tumor-specific response (25, 61). Alternatively, *Salmonella* can induce robust immune responses to APCs via connexin 43 (Cx43) to active CD8+ T cells and enhance anti-tumor immunity. Cx43 is a ubiquitous protein that forms gap junctions in a variety of cell types. *Salmonella* can induce the upregulation of Cx43 and thus enhance gap junction communication, leading to processing of tumor peptides by DCs (25, 64). In the instance of cancer therapy, increased gap junction communication can enhance drug delivery by allowing a drug to penetrate through the complex cellular network seen in tumors (25).

*Salmonella* accumulation in the tumor can also stimulate inflammasome association and tumor apoptosis. Briefly, during inflammasome assembly, a MAMP or damage-associated molecule pattern like LPS interacts with a pattern recognition receptor such as TLR4, leading to activation of transcription factors that encode pro-IL-1β and pro-IL-18 (25, 65, 66). From here activation of various proteins, NLR family pyrin domain containing 3 (NLRP3) being the most famous, forms an inflammasome with or without an apoptosis associated speck-like protein containing a caspase recruitment domain (ASC). ASC recruits pro-caspase 1 or caspase-8 which leads to the maturation and secretion of IL-1β, IL-12, or Gasdermin-D to induce pyroptosis or apoptosis (66). Inflammasome formation can further lead to the activation and interaction of macrophages (65, 66). Macrophages as well as dendritic cells can also be important producers of IL-1β and TNF-α, suggesting that cytokine therapy in addition to *Salmonella* therapy could be of potential value in cancer therapy (67).

Autophagy is another mechanism important for cancer progression. Autophagy is a cellular metabolic recycling process that allows cancer cells to survive the nutrient deprived, hypoxic tumor microenvironment. In pancreatic cancer, autophagy is hyperactive leading to reduced apoptosis and increased proliferation of cancer cells (68–71). The protein kinase B (AKT)/mammalian targets of rapamycin (mTOR) pathway is a significant regulator of autophagy consisting of a cascade of autophagy-related (Atg) proteins encoded by *ATG* genes (69, 71). PDAC cells tend to maintain increased levels of ATG expression leading to higher degrees of basal autophagy activity. This hyperactivity promotes enhanced inflammation and PDAC progression particularly in KRAS-driven tumors (69, 70). *Salmonella* can downregulate AKT/mTOR signaling leading to activated autophagic pathways and thus inhibit tumor growth in certain cancers (25, 71). In pancreatic cancer, the role of autophagy in progression is context dependent, with autophagy generally considered to aid tumor progression once an established tumor has formed. However, greater induction of autophagy has also been shown to promote an autophagic cell death (68). Yet autophagy inhibition induces T-cell and neutrophil infiltration into the TME which causes subsequent pro-tumorigenic inflammation (72). This dual role of autophagy makes the process a challenging therapeutic target. Because of the capacity of *Salmonella* to regulate both apoptosis and autophagy in addition to the aforementioned anti-tumor immune activation, attenuated *Salmonella* is of great interest for bacterial-mediated cancer therapy. A study conducted by Wang et al. showed that combination treatment of hydroxychloroquine, a prominent agent that decreases autophagy activity, and *Salmonella* VNP20009 was associated with longer survival, shorter tumor growth, and enhanced treatment responses in melanoma (73). Therefore, a combination treatment of an autophagy inhibitor like hydroxychloroquine with *Salmonella* could overcome the limitations of standard monotherapy.

### 3.4 Listeria monocytogenes

*Listeria* is a gram-positive intracellular facultative anaerobe which is known for its association with foodborne illness in humans (74). However, over the past few decades, Listeria is being investigated in vaccine development, particularly against cancer. *Listeria monocytogenes* (Lm) elicits strong innate and adaptive immune responses making it a desirable candidate in cancer therapy.

Because *Listeria* localizes within the cytosol, it can directly activate APCs allowing antigen presentation without disseminated infection (27, 74–76). Lm entry into a variety of host cells is complex and involves bacterial cell surface internalins, internalin A (InlA) or internalin B (InlB), which mediate Lm entry via receptor-mediated endocytosis. However, Lm developed mechanisms to evade killing and survive in the cytosol. In the blood, Lm expresses the transcription factor PrfA which activates genes encoding virulence factors like listeriolysin O (LLO), a cholesterol-dependent pore-forming cytolysin similar to SLO discussed previously (56, 74). LLO forms a β-barrel-pore-like structure in the phagosomal membrane allowing Lm to escape into the cytosol. Here, Lm secretes peptides that get degraded by proteosomes and loaded onto MHC class I molecules on APCs resulting in the induction of CD4+ and cytotoxic CD8+ T cell responses (74, 75).

Alternatively, retinoic acid-inducible gene I (RIG-I) recognizes Lm RNA which can lead to the transcription of IFN genes (74). The cyclic GMP-AMP synthase (cGAS) – stimulator of interferon genes (STING) pathway further contributes to transcription of IFN genes (77). Briefly, Lm exposes genomic DNA recognized by cGAS (74, 77). cGAS binds to the dsDNA and forms dimers resulting in the synthesis of 2’3’ cyclic GMP-AMP (cGAMP) which binds to STING and interacts with trafficking factors to pass through the ER-Golgi complex. STING then recruits TANK-binding kinase I (TBK1) which phosphorylates STING and interferon regulatory factor 3 (IRF3) which leads to the expression of type I IFNs to induce pro-inflammatory mediators and priming of CD8+ T cells in the tumor microenvironment (TME) (74, 77). These CD8+ T cells are specific to the tumor associated antigens (TAAs) found within the TME and secrete a variety of cytokines and cytotoxic molecules to induce apoptosis in the tumor (78).

One of the most critical aspects of bacterial mediated cancer therapy is the capability of bacteria to localize specifically in the tumor environment (17). Lm is capable of directly infecting myeloid derived suppressor cells (MDSCs) found within primary tumors. MDSCs in cancer patients can inhibit both T-cell and NK responses. *Listeria* can combat this by infecting MDSCs within the TME and spreading into tumor cells (79, 80). From there, Lm can induce an immunostimulatory phenotype on the MDSCs promoting the production of IL-12 and improving T-cell and NK responses (27, 76, 80). Lm can also directly kill tumor cells by activating NADP+ oxidase and producing reactive oxygen species and activating *Listeria*-specific cytotoxic T lymphocytes (CTL) (27, 75, 76, 81). Kim et al. show that this CTL response contributes to tumor cell regrowth and shrinkage in breast cancer models, making them an important player in cancer mediated therapy (81). The capacity of Lm to both directly and indirectly kill cancer cells allows multiple applications and opportunities for bacterial mediated cancer therapy.

### 3.5 Clostridium novyi

*Clostridium* is a gram-positive, spore-forming, obligate anaerobic bacteria associated with severe human disease. *Clostridium butyricum, C. tetani, C. histolyticum, C. beijerinckii, and C. acetobulyicum* are being observed as anti-cancer agents. One of the best studied *Clostridium* species in cancer therapy is the bioengineered *C. novyi*. Because of the spore-forming capabilities, *Clostridium* is of much interest. Spores can encounter the hypoxic tumor regions, germinate, and become active, leading to tumor regression. Furthermore, the vegetative bacteria cannot spread into normal tissues due to higher oxygen levels between tissues providing tumor specificity and localization to the hypoxic tumor microenvironment (17). Of great interest in cancer therapy, Clostridia strains can be engineered to express cell suicide genes – such as bacterial enzyme cytosine deaminase - or eukaryotic host molecules like TNF-α (61). For example, *C. novyi*- non-toxic (NT) expressing RecA, a protein responsible for DNA repair and maintenance, induces host immunity and the expression of pro-inflammatory cytokines and chemokines leading to tumor regression (61).

Several mechanisms exist for *Clostridium* to provide these tumor destructive forces. *Clostridium* has three lipid-degrading proteins: phospholipase C and two lipases. These enzymes alter the structure of lipid bilayers and membrane permeability of the tumor cells leading to direct cellular cytotoxicity. Phospholipase C can also activate inflammatory responses which can induce anti-tumor immunity through CD8+ T cells. Alternatively, *Clostridium* can modulate innate immune responses. *Clostridium* spores can induce strong inflammatory response involving pro-inflammatory cytokines such as IL-6 which signals immune infiltration by different APCs such as DCs. These DCs can release danger signals that recruit tumor associated antigen specific T cells that induce immunogenic cell death of the tumor cells (17).

## 4 Bacterial mediated cancer therapy: current applications and limitations

Cancer is a complex disease with a variety of cell types and pathological processes participating. The potential of cancer cells to escape host immunity poses a challenge to current standards of treatment. Cancer cells can restrict antigen presentation to APCs, express programmed death-ligand 1 (PD-L1) and secrete immune-suppressive cytokines to negatively regulate T cells, all preventing normal immune cell function (17). Based on the immunomodulatory functions exhibited by the bacteria that are critical to their ability to infect a host that have been discussed in the previous section, bacteria have the promising potential to influence cancer immunity and enhance treatment efficacy.

Since the time of Coley, bacterial mediated cancer therapy has advanced thanks to molecular and technological progress in our understanding of bacteria. Today, bacteria can be used as immunotherapeutic agents or vectors for tumoricidal agents or in combination with other therapies to enhance treatment efficacy (61). Some clinical trials use certain bacteria as forms of cancer therapy. *Salmonella*, for example, acts as a “Trojan horse” for treatment-resistant tumors and *Listeria*-vector based vaccines have been ongoing for the last decade (25, 74). However, despite these achievements, bacterial mediated cancer therapy still has limitations.

### 4.1 Limitations

A major drawback to bacterial mediated cancer therapy is the risk of disseminated infection in immunocompromised patients. Live-attenuated vaccines like BCG or Listeria are potent in their abilities to activate the innate and adaptive immune systems and induce anti-tumor responses (34, 74). Yet, immunocompromised individuals have a propensity for adverse effects. During BCG therapy, some patients require additional prophylactic pharmacotherapies to combat this issue. While this can help reduce the risk of potential infection, the Food and Drug Administration (FDA) is critical about proper antibiotic stewardship (20). Engineering less virulent bacteria could ameliorate this risk but efficacy wanes. Heat-killed Lm strains for example are favorable cancer vaccine candidates for immunocompromised individuals. However, these heat-killed strains do not induce a robust CD8+ T cell response adequate for anti-tumor effects. Killed but metabolically active Lm vaccines, those which contain attenuated pathogens via photochemical inactivation but still retain metabolic activity for sufficient induction of immune responses (82), can counter this problem but are still considered experimental and not in a clinical trial phase (74).

Proper dosage of agents also poses a challenge. The balance between tolerable, efficacious, and toxic is subtle. Many current and proposed bacterial therapies are not long-lasting and require maintenance to be effective. For example, BCG-induced immunity in bladder cancer is short in comparison to the lifelong risk of tumor recurrence with around 30-50% of patients failing to respond to therapy or relapsing within the first five years of treatment (20, 34). To combat this issue, there are efforts to induce a more robust immune response using bacterial components like the BCG cell wall (BCG-CW). However, the BCG-CW has a negative surface charge making interacting with the negatively charged glycosaminoglycans along the urothelium of the bladder poor (34, 35).

Sporulation therapy suffers a similar disadvantage with administration and dosage. Elimination of the phage carrying α-toxin that contributes to the toxicity associated with *Clostridium* makes *C. novyi*-non-toxic (NT) safe for use in cancer patients. Injection of spores into the hypoxic regions of the tumor promotes germination and subsequent regression of the tumor while minimizing the harmful effects of infection. However, if only a partial response is given, the tumors can regrow and become more resistant to *C. novyi*-NT therapy (17). The first human study involving intratumoral injection of *C. novyi*-NT spores (NCT01924689) resulted in three patients developing severe reactions such as sepsis and gas gangrene. Others had manageable toxicities such as rash at the maximum tolerated dose (83). Since the tumor only retains a small fraction of spores, a large dose is necessary; however, this poses the risk of side effects. Moreover, unless spores are delivered to the hypoxic regions of the tumor, the body clears the spores and germination does not occur (17). While this may limit toxicity, the treatment may be less effective in targeting tumors without necrotic areas (16).

Current bacterial mediated cancer therapies rely on a combination of bacteria or bacterial components with chemotherapy or radiation. Combination therapies to such a degree create increased toxicity and side effects which can be detrimental to immunocompromised individuals (17). Therefore, developing ways of delivering effective but safe doses without the need for combination bacteriolytic therapy is necessary to achieve the desirable results without adverse consequences.

### 4.2 Current applications

Although bacterial mediated cancer therapy does pose several limitations as discussed in the previous section, current advances in recombinant technology and bioengineering allow the manipulation of bacteria and bacterial components to combat these issues. Clostridia strains can be engineered to express cell suicide genes – such as bacterial enzyme cytosine deaminase - or eukaryotic host molecules like TNF-α (61). For example, *C. novyi*- non-toxic (NT) expressing RecA, a protein responsible for DNA repair and maintenance, induces host immunity and the expression of pro-inflammatory cytokines and chemokines leading to tumor regression (61).

Engineering attenuated bacteria like *Salmonella* and *Listeria* to be less virulent can manage both dose toxicity and the risk of disseminated infection. The three most recognized attenuated strains in *Salmonella* therapy are VNP20009, AR-1, and ΔppGpp. VNP20009 contains a deleted *purI* gene and a deleted *msbB g*ene to reduce the potential for septic shock and increase antibiotic susceptibility (25). Serovar VNP20009 shows premise with an increased capability to colonize tumors without serious toxicity or side effects shown in a phase I clinical trial (61) in addition to quick clearance from the bloodstream during experimental trial (25).

One of the main mechanisms theorized for the success seen in AR-1 is its ability to stimulate the progression of tumor cells from the G0/G1 phase of the cell cycle to the S/G2/M phase. Because most cytotoxic chemotherapies only recognize and kill tumors in the S/G2/M phase, this can increase the treatment sensitivity in resistant tumors (25).

Serovar ΔppGpp is a *relA* and *SpoT* double mutant defective in ppGpp synthesis, an important protein that plays a role in Salmonella drug susceptibility (25). ΔppGpp has implications in inflammasome activation, cytokine secretion, and heme, iron metabolism, and cytoskeletal structure within the tumor (25, 84). A recent study conducted by Yun et al. shows that tumor tissues from mice treated with ΔppGpp exhibit decreased cytoskeletal components increased iron and heme metabolism suggesting that bacteria proliferation can disrupt tumor tissues while also providing free heme and iron within the tumor environment to enhance bacterial growth (84) allowing for the potential of adequate bacterial colonization without the need for maintenance therapy.

Deletion of *actA* and *inlB* in *Listeria* produces the live-attenuated double-deleted (LADD) strain which is currently the primary candidate for vaccines employed in clinical trials because it exhibits reduced liver toxicity and rapid clearance from the liver and spleen (74). Alternatively, *prfA* gene deletions can severely attenuate Lm strains as seen in the XFL-7 serovar. Partial virulence can be restored to an immunogenic level with the addition of a plasmid encoded PrfA cloned with a tumor-associated antigen (TAA). The *Δdal/Δdat* (Lmdd) strain, deficient in the genes necessary to build peptidoglycan and dependent on alanine co-administration, can also regain partial virulence with the use of a plasmid without the addition of alanine. Both Lmdd and XFL-7 strains are currently being used in clinical trials (74).

Several combination therapies have been proposed using these modified organisms. For example, *Listeria* combined with the autophagy inhibitor chloroquine shows inhibited tumor growth, increased accumulation of Lm within the tumor, and higher incidences of cell apoptosis (85). Currently, the M.D. Anderson Cancer Center in collaboration with BioMed Valley Discoveries, Inc and Merck Sharp & Dohme LLC is investigating the antitumor effects of combination treatment of pembrolizumab and *C. novyi*-NT (NCT03435952) followed by management of Doxycycline on malignant neoplasms of the breast, digestive organs, urinary tract, head and neck, and genital organs (86). While these show promise, there is limited evidence suggesting increased efficacy in metastatic PDAC patients.

As discussed previously, combination therapies pose a risk for higher toxicity. To combat this issue, fusion-based vaccines are in development. Fusion-based vaccines utilize a bacterial protein fused with a TAA to enhance delivery of the TAA into the TME subsequently stimulating APC maturation and pro-inflammatory cytokine secretion and inducing a more robust anti-tumor response. One of the most notable fusion-based vaccines, CRS-207, uses the *Listeria* protein ActA fused with the TAA mesothelin. Overexpression of mesothelin is associated with many different cancer cell types including pancreatic cancer (74). Although the initial phases of CRS-207 proved safe and efficacious for PDAC patients, during phase II after receiving priming-boosts with GVAX and cyclophosphamide (Cy), patients showed no differences in mesothelin-specific CD8+ T cells responses in comparison to patients receiving Cy/GVAX alone or gemcitabine alone (74, 87). Currently, additional trials are attempting further prime-boosting using checkpoint inhibitors like αPD-1 and αCTLA-4 as well as indoleamine dioxygenase inhibitors (74). However, at this current time, there is little evidence suggesting the efficacy in treatment resistant patients.

### 4.3 Opportunities for enhancement

Many new bacterial therapies have been proposed within the last decade with several showing potential in clinical trials. Still with the proposed risks associated with bacterial mediated cancer therapy, there is a need to enhance efficacy and improve overall patient response. Nano delivery of bacterial components, TLR receptors, and enhancing the microbiota are just a few routes of improvement.

Nanoparticles (NP) are gaining recent recognition for their ability to ameliorate toxicities and enhance drug delivery. Evidence indicates that NPs can increase drug delivery into the tumor while reducing bacterial toxicity on host cells (88). One group utilizes an R8-liposome which acts as an enveloped virus to improve BCG-CW delivery. This provides successful inhibition of tumor growth because the R8-liposome has a cell penetrating peptide that promotes the entry of BCG-CW into the cytoplasm of the bladder cancer cells (20, 35). In PDAC, NPs are designed as liposomes, micelles, and more recently mesoporous silica (MSNPs) to deliver drugs to the pancreatic tumor microenvironment (88, 89).

Currently, only a few drugs exist using nanotechnology. Albumin-bound paclitaxel (PTX) nanocarrier (Abraxane) is the first new drug for PDAC in over a decade. PTX improves the overall efficacy of gemcitabine by inhibiting the expression of cytidine deaminase in the stroma, thus preventing gemcitabine degradation (90). Meng et al. show that the effects of PTX and gemcitabine could be enhanced when packaged in a mesoporous silica nanoparticle (MSNP). Co-delivery of PTX and gemcitabine via MSNP exhibits more pronounced tumor shrinkage and inhibition of tumor growth with little evidence of toxicity (90). Liposomal irinotecan (Onivyde) was approved by the FDA in 2015 for use in metastatic PDAC. A combination treatment of Onivyde and 5-fluorouracil(5-FU)/Leucovorin shows increased survival averages of two months with a three-month delay in tumor growth in comparison to individuals who receive 5-FU/Leucovorin alone (88).

However, liposomal formulations have limitations. A major issue is systemic drug leakage and a loss of selectivity over time due to exposure of biomolecules in fluids like the blood (88, 89). These molecules form a biomolecular corona that has the capacity to reduce targeting capabilities of the NP (91). Liu et al. show that by loading irinotecan across a coated lipid bilayer in a MSNP carrier there is significantly less drug leakage and therefore less toxicity in comparison to the liposomal carrier. Moreover, because there is less drug leakage, there is a higher concentration of drug delivered to the tumor, thus improving drug efficacy (89).

Still, chemotherapy has toxicity and potential harsh side effects, impacting quality of life and leading to dose reductions and treatment delays. While bacteria can pose a threat to immunocompromised individuals, isolated bacterial proteins could produce positive results without these types of detrimental effects. Listeria and Salmonella both have intrinsic capabilities to kill cancer cells directly (27, 74, 76). Therefore, packaging a protein like flagellin or a specific bacterial antigen in a tumor could induce a cytotoxic T cell response directed specifically at the tumor without the toxicities associated with cancer drugs. A bacterial antigen in a NP carrier could also mitigate the risk of disseminated infection as active bacteria are not being injected into a tumor.

Another alternative could be the use of TLR agonists. TLR agonists may be beneficial in a combination treatment with BCG, for example. TLRs play a role in the innate and adaptive immune responses in urothelial cells with reduced levels suggested in bladder tumors. The addition of a TLR agonist could prevent relapse associated with BCG treatment (20). Because there are a variety of TLRs and associated pathogen associated molecular patterns (PAMPs), like flagellin and TLR5 (27), TLR agonists could provide customization towards the bacteria in use and corresponding desired anti-tumor response.

The tumor microbiome is gaining recognition for a potential role in influencing responses to cancer immunotherapy. The microbiome is found to be a predictive marker for immune checkpoint blockade (ICB) response (92–94). Responders to treatments not only show differences in microbiota composition in comparison to non-responders but they also show immune profiles associated with increased anti-tumor immunity (92, 93). Several studies demonstrate that in melanoma, patients who had positive responses to anti-PD-L1 therapy had a higher abundance of the members of the *Ruminococcaceae* family, as well as species of *Bifidobacterium longum* and *Enterococcus faecium*. Moreover, a bacterial transfer from these patients into germ-free mice generated higher anti-tumor immune profiles similar to the responders and better responses to anti-PD-L1 (92, 95). A phase 1 clinical trial (NCT03353402, Sheba Medical Center) conducted by Baruch et al. demonstrated that following fecal microbiota transplantation, recipients had an up-regulation of immune-related gene sets related to IFN-γ signaling and Th1-type immune responses (96, 97). These patients also showed an abundance of bacteria taxa that are associated with responses to anti-PD-1 and CD8+ T cell activation. These results suggest that fecal microbiota transplantation could be able to affect immunotherapy response such as that to anti-PD-1 therapy (97).

However, there is evidence to suggest that probiotic bacteria can trigger both favorable and unfavorable responses to ICB. Many patients take probiotics for health benefits. Several probiotic strains described previously are being investigated for anti-cancer benefits. Lactobacillus, for example, can exert immunomodulatory effects like increased production of IL-2 and IL-12, antioxidant activity like production of superoxide dismutase and glutathione, and preventing DNA damage, thus, leading to decreased inflammation and tumor growth. There is also evidence suggesting a role in regulating the metabolic activity of indigenous microbiota which can modulate host immune responses and inflammatory pathways (98).

One of the better studied mechanisms is the ability of *Lactobacillus* and *Bifidobacterium* to augment NK cell activity (98, 99). A study conducted by Ogawa et al. demonstrates that oral administration of *L*. *casei ssp. casei* in a combination treatment with dextran increases NK cell activity in healthy individuals. There are also significant increases in IL-12 production suggesting that dietary supplementation of Lactobacillus could enhance NK cell activity and lead to lower cancer risk, suggesting a protective advantage to individuals predisposed to cancer (98, 99).

Probiotics can also affect the activities of APCs and subsequent T cell activation. Probiotic bacteria have a variety of MAMPs on the bacterial surface. Lipoteichoic acid, LPS, and flagellin can interact with different TLRs on the surface of DCs which can modulate T cell polarization and lead to antitumor effects (99). Sivan et al. found that DCs isolated from tumor-bearing mice in a melanoma model had an upregulation of genes involved in CD8+ T cell activation, DC maturation, chemokine-mediated recruitment of immune cells to the TME, and type 1 interferon signaling after oral administration of *Bifidobacterium*. results were comparable to mice receiving a programmed cell death protein 1 ligand 1 (PD-L1) therapy, a checkpoint blockade. The combination treatment of oral *Bifidobacterium* and PD-L1 nearly abolished tumor growth (94).

Yet, diet can have a significant impact on the benefit of probiotic usage. A study conducted by Spencer et al. compared ICB responses in mice receiving high-fiber or low-fiber diets with or without probiotic supplementation. Mice receiving high-fiber and no probiotic usage achieved the best responses. However, mice receiving low-fiber diets or probiotic supplementation alone had poor ICB responses with lower microbial diversity and suppressed intratumoral T cell responses (100). This suggests that while probiotic bacteria can provide a protective effect in some cases, tailored probiotics composed of the specific strains from treatment responders could benefit more than a typical over-the-counter probiotic supplement for patients receiving ICB therapy.

## 5 Perspective on bacterial immunotherapy of PDAC

While bacterial mediated cancer therapy provides promise in many areas of cancer research, consistent evidence for success in PDAC treatment is still nominal. While several vaccines show substantial premise, CRS-207 for example (74, 87), challenges persist. Still, existing therapies create insight on feasible ways to mitigate cancer therapy-associated toxicities and enhance treatment efficacy.

The importance of the microbiome is increasingly recognized across various diseases and cancer. Insights into the role of the microbiome in PDAC may unveil potential bacterial therapeutic strategies. Currently, there is substantial evidence suggesting that the microbiome plays a crucial role in the development of PDAC. Microbial compounds like LPS, CpG DNA, and lipopeptides can induce inflammation leading to immune suppression by the binding of these pattern recognition receptors and activating TLRs (101). Pushalkar et al. show that bacterial ablation in the cancerous pancreas leads to immunogenic reprogramming in the TME and a shift to an immunostimulatory state. However, reconstitution with bacteria from PDAC hosts leads to the activation of select TLRs (TLR2 and TLR5) in macrophages which contributes to PDAC tumor growth and innate and adaptive immune suppression (102). Activation of TLRs can also help to maintain inflammation during carcinogenesis which can trigger oncogenic KRAS mutations. While KRAS is a common mutation among PDAC tumors, inflammation caused by LPS can still contribute to KRAS activation, further advancing PDAC development (101). Several studies show that PDAC patients have a distinct microbiota that differs from healthy individuals (102–105). Moreover, the microbiome and its associated metabolic pathways differs among long-term versus short-term PDAC survivors (97, 105). A study conducted by Huang et al. demonstrates that long-term survivors have a more diverse intratumor microbiome which is associated with more favorable patient outcomes (106). By transferring bacteria from healthy individuals into cancer patients, the immune system may shift to a more tumor-suppressing phenotype directly enhancing the cytotoxic effects of CD8+ T cells or activating mechanisms such as STING signaling or NK cell activation (102, 105–107).

Intratumor bacteria can also metabolize chemotherapeutic drugs such as gemcitabine. Since gemcitabine is part of the standard care for PDAC patients, preventing degradation is essential to achieve positive outcomes. Geller at al. show that a large proportion of human PDAC samples are positive for Gammaproteobacteria which express cytidine deaminase. Cytidine deaminase directly contributes to treatment resistance by degrading gemcitabine into its inactive form (103, 104). While co-administration with ciprofloxacin seems to abrogate these effects (103), organizations such as the FDA are critical about antibiotic stewardship (20).

Therapy involving oral administration of probiotic bacteria might be more effective and safer in patients. Oral administration of probiotics can enhance the efficacy of gemcitabine (33). Chen et al. demonstrate in *LSL-Kras ^G12D/–^* Pdx-1-Cre transgenic mice that oral administration of probiotics and intraperitoneal injection of gemcitabine reduce pancreatic intraepithelial neoplasia formation as well as lower liver toxicities associated with chemotherapy. The finding of reduced liver toxicities suggests that probiotic supplementation could ameliorate some of the harsh side effects of chemotherapy (33).

However, the majority of these studies are proposed in mouse models with little research showing the efficacy in humans. These mouse studies could provide the framework for future clinical trials. Further research identifying mechanisms and specific endogenous microbial populations in PDAC patients is also necessary to determine the efficacy of probiotic administration and how modulation of the microbiome can benefit patients.

The TME of PDAC patients is complex. Aside from the array of intratumor bacteria, the stroma is comprised of several cell types like fibroblasts and pancreatic stellate cells as well as immune cells, collagen, and other ECM-related proteins (3, 108). Cancer-associated fibroblasts (CAFs) are one of the most important components of the ECM (108). Several bacteria harbor mechanisms that target fibroblasts and collagen as discussed previously. Scl1 from GAS can support the interaction of GAS and collagen-binding integrins allowing internalization into human cells (38) as well as attachment to the ECM deposition due to CAFs (39). Modulating GAS as a vector to deliver cancer drugs could be beneficial. Alternatively, expressing Scl1 on a less virulent lactic acid bacteria, lactobacillus for example, may provide enhanced drug delivery while minimizing the risk of side effects and infection. However, varying populations of CAFs exist within pancreatic tumors (109, 110). Inflammatory CAFs (iCAFs) are the primary population seen in hypoxic tumor regions. Hypoxia can promote cues for inducing the different populations of CAFs and thus regulate the heterogeneity in PDAC tumors (110). Future studies looking at how GAS and other bacteria interact with the different CAF populations could show a new mechanism for avoiding resistance in stroma-targeting therapies. Furthermore, understanding the specific role and regulatory elements expressed by these CAF populations could provide new targets for future cancer drugs.

Since NETs have been implicated in pancreatic cancer progression through several different mechanisms, targeting NETs is a promising avenue for future research. Patients with higher neutrophil infiltration and NETs exhibit poorer prognosis and disease-specific survival (111). NETs can promote metastasis, induce the epithelial to mesenchymal transition, and directly induce tumor cell proliferation (50, 51). Since many bacteria can evade NETs and prevent NET degranulation, using bacteria to target NETs may enhance patient prognosis. GAS employs a variety of mechanisms to prevent NET formation and degradation while enhancing NET resistance (112). SLO toxin can prevent neutrophil degranulation and elicit further protective immunity if administered in subcytotoxic concentrations (56). The Sda1 DNase found in GAS can also play a role in NET degradation and innate immune evasion (55, 60, 113). While both SLO and Sda1 can contribute to virulence (56, 60, 113), expressing the proteins in less virulent bacteria could potentially provide NET inhibition without the risk of severe disease. Using a TAA-fusion model like that in Listeria (74) could also show promise by providing NET inhibition and enhancing drug delivery. Fusing a non-pathogenic, structurally modified version of the Sda1, SLO, or Scl1 to a TAA like mesothelin, a prominent TAA in PDAC (74, 87) could be beneficial in inducing anti-tumor responses. Two clinical trials, NCT01018784 and NCT00711191 by Eisai Inc. and Hoffman-La Roche respectively, used an anti-mesothelin monoclonal antibody MORAb-009 as treatment for PDAC. However, these studies showed suboptimal results (114, 115). A comparison study between mesothelin monoclonal antibodies and TAA-fusion treatments could provide insight on how to improve current immunotherapies using similar techniques.

A more direct approach to bacterial therapy without the need for vaccination or combination with drug delivery is through radionuclide therapy. Targeted radionuclide therapies using radiolabeled antibodies to TAAs in PDAC have been assessed in the last decade demonstrating safety and efficacy. Multiple clinical trials show progress using radiolabeled 90Yttrium and 177Lutetium in pancreatic neuroendocrine tumors (NCT02230176 - Gustave Roussy, NCT05568017 – European Institute of Oncology, NCT02358356 – Australasian Gastro-Intestinal Trials Group) (116–119). Clivatuzumab, PAM4, is a monoclonal antibody in clinical trial with a high specificity for PDAC targeting mucin species which contribute to a tumorigenic environment by enhancing cancer cell immune evasion (120, 121). Ocean et al. demonstrate that a combination of single-dose 90Yttrium-labeled-humanized-clivatuzumab-tetraxetan with Gemcitabine can increase the median survival of patients with pancreatic ductal carcinoma but seven to eleven months if receiving repeated cycles at higher immunotherapy doses (NCT00603863) (119, 122, 123). Although 42% of patients showed stabilization as the best response, there was still a need for multiple cycles of therapy and high amounts of dosing to gain partial responses (123).

Quispe-Tintaya et al. reported the first bacterial radioactive treatment of pancreatic cancer using Rhenium-188 coupled to attenuated *Listeria* to generate radioactive *Listeria* (RL). Because of the immunosuppressive TME of PDAC and metastatic tumors, attenuated Listeria can efficiently be cleared in normal tissues but not the TME. When administered in multiple injections at low doses, RL demonstrated dramatic decreases in the number of metastases (90%) in a Panc02 metastatic model (119, 124).

As mentioned previously, virtually all PDAC tumors harbor KRAS mutations (1, 9, 11). Chandra et al. develop a model using 32-phosphorous (32P) incorporated into Listeria monocytogenes to target KPC tumors, a tumor cell line that contains both KRAS and p53 mutant alleles, and metastases. Engineered *Listeria*-32P kills tumor cells through the induction of ROS by *Listeria* and 32P-induced ionizing radiation. Therapeutic treatment with *Listeria*-32P not only reduced pancreatic cancer at early and late stages but it also had minimal side effects (79). Therefore, utilizing a KPC cell line could benefit further studies in PDAC to give a better clinical translation. Moreover, using radionuclide bacterial therapies with other organisms like Salmonella, another intracellular pathogen, could be of interest.

## 6 Conclusions

PDAC is a lethal disease with a five-year survival rate of only 10% (2, 10). The standard treatment consists of gemcitabine or FOLFIRINOX and surgical resection (2). Yet, these still show only slight improvement for patient outcomes. New drugs like nab-paclitaxel (90) or liposomal irinotecan (88) show promise, but resistance mechanisms and toxicities are still problematic. Therefore, developing alternative therapies is critical.

This review summarizes the history, current applications, and limitations of bacterial mediated cancer therapy. While understudied in PDAC, bacterial mediated cancer therapy shows much promise in a variety of cancers. Therefore, additional research is needed to apply ongoing bacterial-mediated therapeutic strategies in PDAC. While immunotherapy can trigger an immune response against tumor-specific antigens, these immunotherapy-based strategies are still less effective in PDAC due to a unique, immunosuppressive TME with vast heterogeneity (2). By colonizing tumors, bacteria can stimulate the immune system to target bacterial antigens within the tumor as opposed to tumor antigens themselves and ultimately lead to IFN-γ and Th1 responses that induce acquired immunity in a tumor-specific manner (61). Moreover, many bacteria are facultative or obligate anaerobes allowing them to combat the resistance mechanisms seen in hypoxic tumors (25), overcoming a significant hurdle to effective therapies.

While no therapy is without limitations, bacterial mediated cancer therapy provides much promise in overcoming the challenges of standard PDAC treatment regimens. As chemotherapeutic agents continue to advance, the research conducted by Coley and others cannot be discounted. Bacteria allow researchers the opportunity to manipulate and exploit an array of adaptations to enhance cancer drug delivery as well as therapeutic efficacy. Perhaps, bacteria are a missing piece in the puzzle for a cancer cure.

## Author contributions

All authors contributed to conceptualization of review. EH wrote the first draft of manuscript. All authors contributed to the article and approved the submitted version.
